# Pembrolizumab for metastatic melanoma in a renal allograft recipient with subsequent graft rejection and treatment response failure: a case report

**DOI:** 10.1186/s13256-017-1229-z

**Published:** 2017-03-19

**Authors:** Vineet Kwatra, Narayan V. Karanth, Kelum Priyadarshana, Michail Charakidis

**Affiliations:** 1 Medical Oncology Department, Alan Walker Cancer Centre, Royal Darwin Hospital, 105 Rocklands Drive, Tiwi, NT 0810 Australia; 2 NT Renal Services, Royal Darwin Hospital, Darwin, NT Australia; 3 Flinders University, Adelaide, SA Australia

**Keywords:** Melanoma, Checkpoint inhibitors, Transplant, Toxicity, Case report

## Abstract

**Background:**

Transplant patients were excluded from the pivotal phase III trials of checkpoint inhibitors in metastatic melanoma. The efficacy and toxicity profiles of checkpoint inhibitors in this cohort of patients are not well described. To the best of our knowledge, this is the first case report of a renal transplant patient with stage IV melanoma treated with a programmed cell death protein 1 checkpoint inhibitor that led to both treatment failure and renal graft rejection.

**Case presentation:**

We present a case of a 58-year-old white man with a long-standing cadaveric renal transplant who was diagnosed with a B-Raf Proto-Oncogene, Serine/Threonine Kinase wild-type metastatic melanoma. He was treated with first-line pembrolizumab but experienced subsequent graft failure and rapid disease progression.

**Conclusions:**

This case highlights the risks associated with the administration of checkpoint inhibitors in patients with a renal transplant and on immunosuppressive therapy. More specifically, it adds to the literature indicating that, compared with the cytotoxic T-lymphocyte-associated protein 4 inhibitor ipilimumab, anti-programmed cell death protein 1 agents are more likely to lead to renal graft failure. Additionally, these novel immunotherapeutics may be ineffective in transplant patients; therefore, clinicians should be very aware of those risks and carefully consider selection of agents and full disclosure of the risks to their patients.

## Background

Programmed cell death protein 1 (PD-1) immune checkpoint inhibitors have been shown to significantly improve overall survival among patients with many solid malignancies, in particular patients with metastatic melanoma and stage IV small cell and non-small cell lung cancer [1, 2]. Transplant patients were excluded from the pivotal phase III trials of immune checkpoint inhibitors; therefore, the efficacy and toxicity profiles of these agents in this cohort of patients are not well described. Furthermore, there is a potential increased risk of acute graft rejection owing to the activation of T cells from PD-1 inhibition and risk of cancer treatment failure due to the concurrent use of immunosuppressive therapy required to maintain transplant organ function. In this report, we describe a case of a renal transplant patient with stage IVB-Raf Proto-Oncogene, Serine/Threonine Kinase (BRAF) wild-type melanoma who was treated with first-line pembrolizumab (anti-PD-1 agent) that led to subsequent graft rejection and rapid disease progression.

## Case presentation

A 58-year-old white man received a cadaveric renal allograft in 2001 for end-stage renal disease secondary to immunoglobulin M nephropathy. The human leukocyte antigen (HLA) compatibility of donor to recipient showed a mismatch of two antigens with peak panel reactivity antibody of 9%. The patient had been receiving tacrolimus 1.5 mg twice daily and mycophenolate mofetil (MMF) 500 mg twice daily over the past 13 years without any evidence of chronic rejection. The transplanted kidney had been functioning well with baseline serum creatinine <100 μmol/L. The patient’s other significant comorbidities included hypertension, multiple resected squamous cell skin cancers, hepatitis C, and treated latent tuberculosis.

**Fig. 1:**
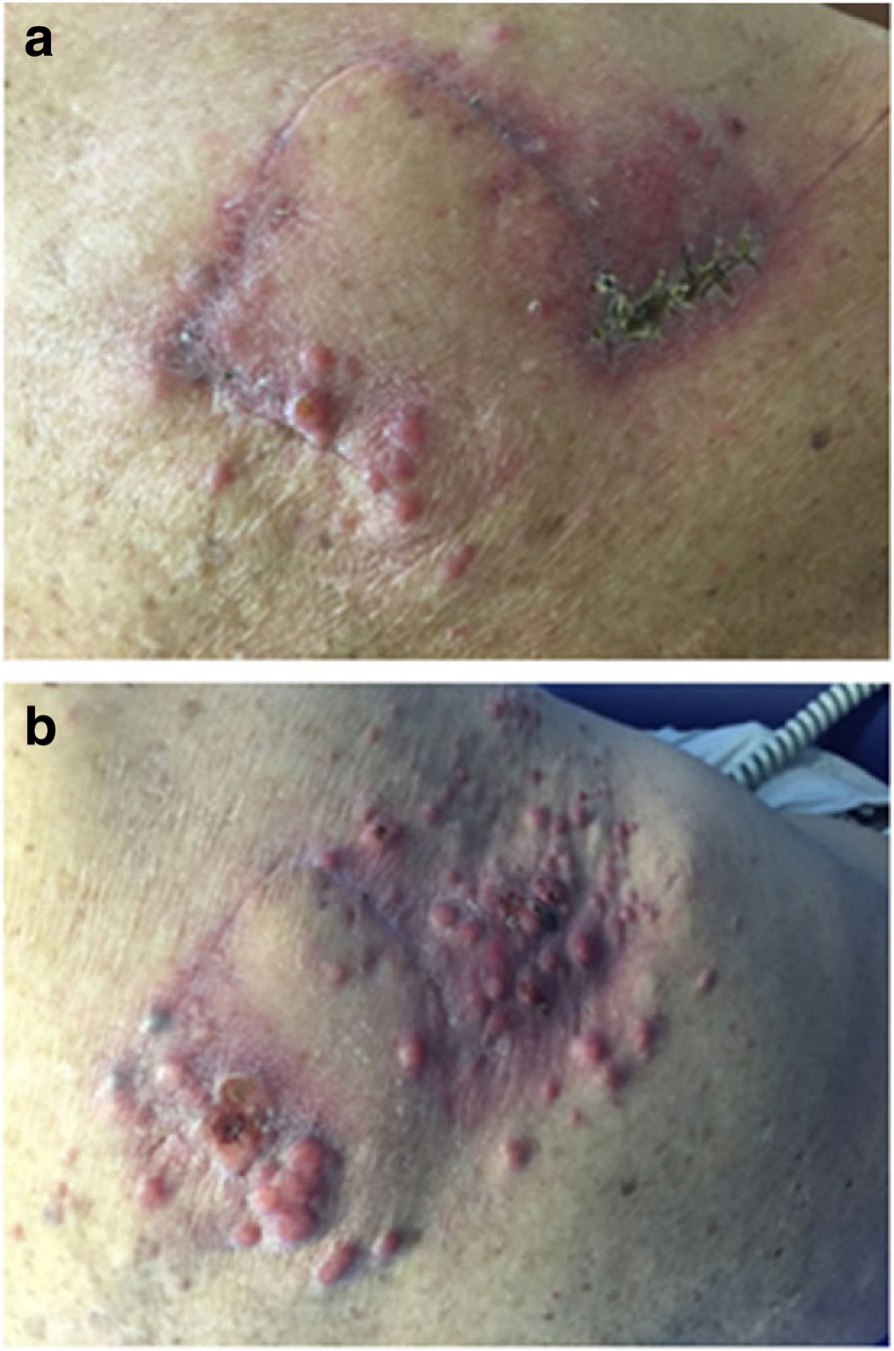
**a** Fungating melanoma over the right scapula at diagnosis. **b** Progressive disease of cutaneous lesions after two cycles of pembrolizumab

**Fig. 2:**
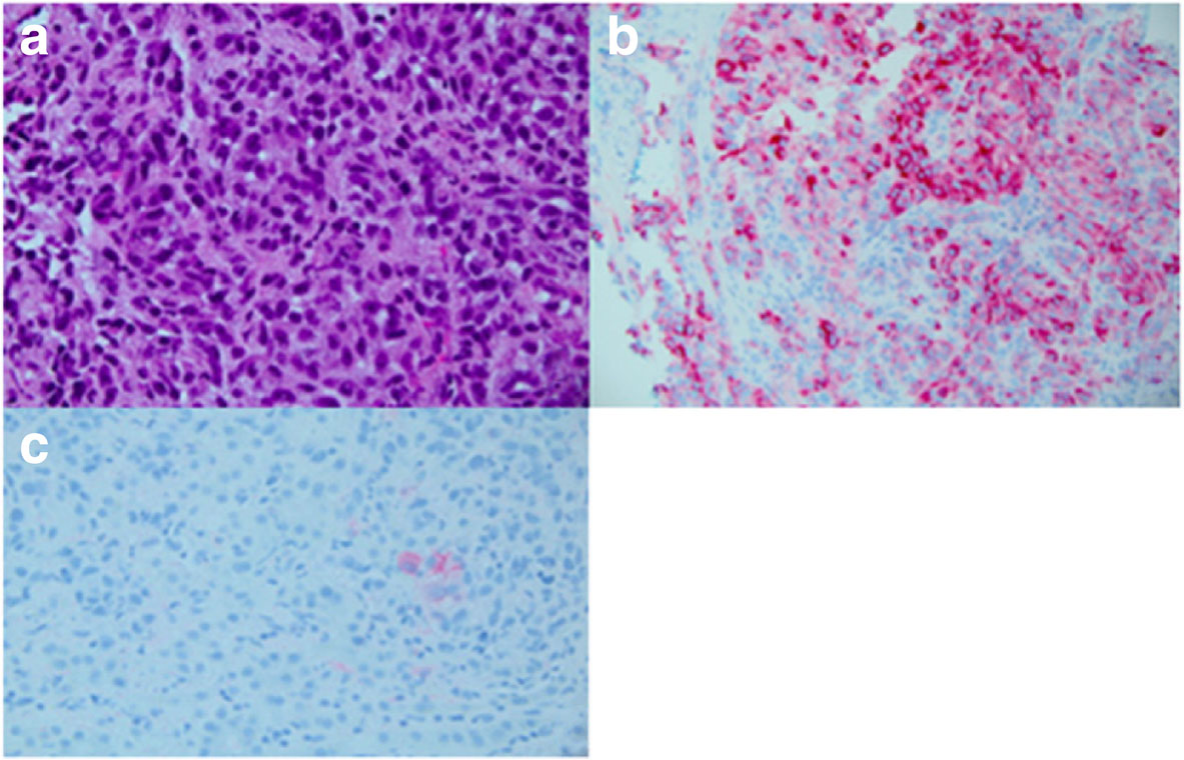
Malignant melanoma visualized by immunohistochemistry. **a** High-power view. **b** Melan-A stain. **c** S100 stain

The patient noticed a rapidly enlarging, fungating skin lesion over the right scapula (Fig. 1a) and complained of unintentional weight loss of 3 months’ duration. An excisional biopsy was performed, which showed an ulcerated nodular melanoma of 21-mm thickness, Clark level V. The patient’s immunohistochemistry was positive for homatropine methylbromide 45 (HMB-45), S100, and melan-A (Fig. 2), and a molecular study showed wild-type BRAF status. Computed tomography-based staging revealed multiple liver metastases, an L4 lytic lesion, and left hilar and porta hepatis lymphadenopathy. A liver biopsy was performed, which also confirmed metastatic melanoma. Tacrolimus and MMF were stopped. The patient was started on azathioprine 100 mg daily and everolimus 0.5 mg twice daily prior to commencement of pembrolizumab 2 mg/kg every 3 weeks. The reason for modification of immunosuppressive drugs was to maximize the treatment effect of the anti-PD-1 inhibitor. Prior to treatment, the patient and his family were informed about the potential risks of graft failure and progression of melanoma.

**Fig. 3:**
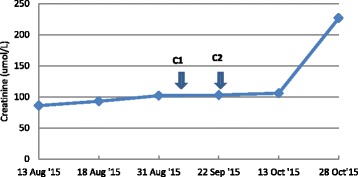
Rise in creatinine in relation to cycle 1 and cycle 2 (C1 and C2, respectively) of pembrolizumab

After two cycles of pembrolizumab, the patient’s condition continued to decline rapidly, with reduced performance status and an increase in the size of subcutaneous nodules over his right scapular area (Fig. 1b). His renal function worsened rapidly with a creatinine level >200 μmol/L (Fig. 3). He declined the options of hemodialysis and renal biopsy owing to his poor prognosis. His liver enzyme and electrolyte levels were within normal ranges, and his chronic normocytic anemia was unchanged with a hemoglobin level of 106 g/L. He had mild neutrophilia (9.6 × 10^9^/L). His C-reactive protein level was 77 mg/L. He was transferred to a hospice so he could receive best supportive care, and he died there 3 days later.

## Discussion

**Fig. 4:**
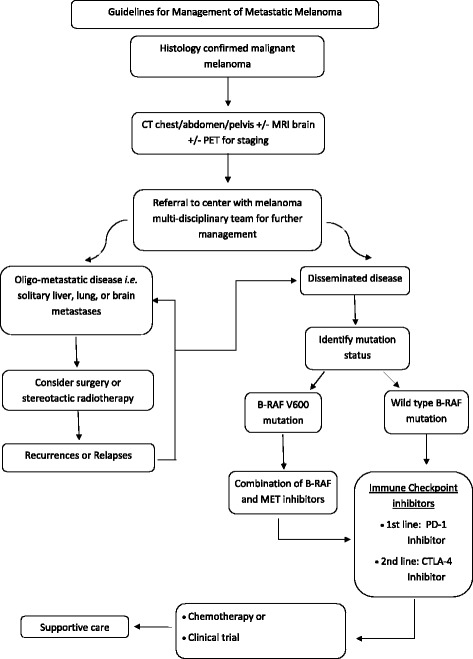
Treatment guideline algorithm for metastatic melanoma. *CT* Computed tomography, *CTLA-4* Cytotoxic T-lymphocyte-associated protein 4, *MET* Mesenchymal-epithelial transition factor, *MRI* Magnetic resonance imaging, *PD-1* Programmed cell death protein 1, *PET* Positron emission tomography

To the best of our knowledge, this is the first report describing first-line use of a PD-1 inhibitor in a renal transplant patient for the management of BRAF wild-type metastatic melanoma without prior immunotherapy, targeted therapy, or chemotherapy. To the best of our knowledge, this is also the first report describing a synchronous graft rejection and treatment failure with rapid and fatal disease progression. There were two recent published case reports on PD-1 inhibitors causing renal allograft rejection [3, 4]. In these two reports, patients had BRAF wild-type metastatic melanoma managed with a first-line cytotoxic T-lymphocyte-associated protein 4 (CTLA-4) inhibitor (ipilimumab) with maintenance of the graft while on ipilimumab. After progression, the treatment was switched to a PD-1 inhibitor, with subsequent graft rejection requiring hemodialysis. One of the patients was able to achieve disease control following six cycles of a PD-1 inhibitor. Furthermore, there are two case reports on renal transplant patients with stage IV melanoma who were treated with ipilimumab and had good disease response and graft preservation [5]. A general treatment guideline algorithm for metastatic melanoma is shown in Fig. 4.

T-cell activation or tolerance to self-antigen depends on the balance between costimulatory and coinhibitory signaling [6]. Evidence has shown that the PD-1 and programmed death-ligand 1 (PD-L1) pathway is implicated in transplant tolerance and prevention of chronic allograft rejection [7]. In addition, the PD-L1 receptor is most prominent in renal tubules and highly regulated in renal transplant recipients. Therefore, it plays an important role in impairing T-cell response to the transplant organ [8]. Early data showed that blocking PD-L1, and not PD-L2, can accelerate graft rejection of a major histocompatibility complex class II mismatch allograft [9]. This may explain the currently available data demonstrating the ability to maintain the allograft when treated with anti-CTLA-4 inhibitors but rejection of the transplanted tissue when treated with anti-PD-1 agents [3, 4].

Tacrolimus is the backbone immunosuppressant for use in renal transplant patients because it has been shown to be associated with long-term graft survival [10]. It acts by inhibiting T-cell signal transduction. The common perception in management of transplant recipients with a new diagnosis of melanoma is to reduce or change immunosuppressants depending on several factors, including age, HLA mismatch, and prior history of rejection. Azathioprine and mammalian target of rapamycin inhibitors are both reasonable step-down options in long-term recipients when attempting to maintain graft function [11]. Despite this, there is inadequate data to help select the most appropriate immunosuppressant and its interaction with immune checkpoint inhibitors. Acute allograft dysfunction in this setting is most likely to be related to acute cell-mediated rejection and acute tubular necrosis. Lipson *et al*. [12] reported a histologically proven case of acute cell-mediated renal allograft rejection that occurred about 2 months after administration of PD-L1 inhibitors without the associated antibodies that participate in rejection. Although their patient had cutaneous squamous cell carcinoma, a similar mechanism for transplant rejection can be reasonably assumed in our patient.

## Conclusions

Renal transplant patients with stage IV melanoma are more likely to maintain their graft and have a response if treated with ipilimumab than if they are treated with anti-PD-1 agents [5]. PD-1 inhibitors may result in disease response, but they are more likely to threaten the transplanted renal tissue with rejection [3, 4]. Therefore, ipilimumab should be considered as first-line therapy in renal transplant patients with stage IV melanoma requiring treatment with immunotherapy. As highlighted by our patient’s case, there is always a risk of graft failure and disease progression in kidney transplant recipients on immunosuppressive therapy who are treated with checkpoint blockade. These patients should be made aware of this risk.
